# 多原发肺癌的诊断和处理策略新进展

**DOI:** 10.3779/j.issn.1009-3419.2016.05.12

**Published:** 2016-05-20

**Authors:** 海法 郭, 屠阳 申

**Affiliations:** 200039 上海，上海交通大学附属胸科医院/上海市肺部肿瘤临床医学中心 Shanghai Chest Hospital, Shanghai Jiaotong University, Department of Shanghai Lung Tumor Clinical Medical Center, Shanghai 200039, China

**Keywords:** 多原发肺癌, 发病机制, 诊断, 处理策略, Multiple primary lung cancer, Pathogenesis, Diagnosis, Management strategies

## Abstract

原发肺癌（multiple primary lung cancer, MPLC）的发病率呈逐年上升趋势，但关于MPLC的发病机制、诊断、鉴别诊断和临床处理策略仍存在诸多争议。本综述将对目前关于MPLC的发病机制、诊断标准、鉴别诊断和处理策略方面的研究进展做一个回顾，旨在为该病的诊疗提供理论基础。

## Abstract (en)

Although the morbidity of multiple primary lung cancer (MPLC) has been increasing year by year, it is still controversial about pathogenesis, differential diagnosis and management strategies of MPLC. This review provides a snapshot of the main progress of pathogenesis, differential diagnosis and management strategies of MPLC.

多原发肺癌（multiple primary lung cancer, MPLC）是指在同一患者肺内同时或先后发生两个或两个以上原发性恶性肿瘤，以诊断时间间隔6个月为界，分为同时性MPLC（synchronous MPLC, sMPLC）和异时性MPLC（metachronous MPLC, mMPLC）。1924年，Beyreuther^[1]^第一次报道同时患有两个彼此独立肺癌的病例，并首次引入了多原发肺癌的概念。以往认为，MPLC是一种较少见的疾病，但近些年其发病率呈不断上升趋势，上限增加近3倍之巨，令人瞩目。

## 1 MPLC的流行病学

整体来说，近年MPLC的发病率呈不断升高趋势，2000年之前报道的发病率为0.2%-3.4%^[2, 3]^，而2000年之后则为0.3%-8.0%^[4-7]^。此外，国内外MPLC发病率也存在差异，国外报道为0.20%-8.00%^[3, 5]^，而国内为0.3%-3.44%^[1, 4]^，低于国外报道。在可手术的Ⅰ期-Ⅲ期非小细胞肺癌（non-small cell lung cancer, NSCLC）患者中，大约有16%的术前影像学检查发现存在多结节病灶^[8]^，其中1%-8%的多结节病灶为MPLC^[9]^。在第二原发肺癌的组织学分布中，Pillai^[10]^报道，腺癌最多（40.7%），其他类型次之（30.1%），余为鳞癌（25.6%）和大细胞癌（1.8%）；近期另一项报道^[11]^腺癌占86.49%，这可能与肺腺癌发病率升高有关。Chang^[12]^的研究认为，两肺的上叶容易发生多原发肺癌，并且多个病灶的病理学类型相同的患者占50%-70%。

## 2 MPLC发病原因与发病机制研究

### 2.1 区域性癌化

1953年，Slaughter^[13]^通过对口腔多原发鳞癌的研究提出了“区域癌化”，认为口腔多原发癌由多灶的癌前病变在相同的致癌因素作用下相互独立发展而来。数年后，Strong^[14]^将“区域癌化”这一学说扩展到整个呼吸系统，认为致癌原介导的DNA改变存在于整个呼吸道粘膜，支气管肺泡上皮可能广泛异型增生，其中某些高级别的异型增生细胞首先癌变，随着时间的推移，还可能有其他异型细胞相继癌变，形成多原发肺癌。此后，“区域癌化”被多位研究者所证实。Sikkink^[15]^对一例诊断为左主支气管原发性鳞癌的男性患者进行了详细研究，首先对该患者手术切除的左全肺标本的各级支气管间断每隔1 cm进行取样，对所取到的样本进行了7个微卫星位点杂合性缺失分析，并检测了每个样本蛋白p53和Cyclin D1的表达水平。研究结果表明，肿瘤内存在等位基因杂合性缺失，同时左肺样本中83%组织学正常的支气管粘膜亦存在等位基因杂合性缺失，且不同位置粘膜的缺失方式存在差异；正常的上皮粘膜也表达p53和Cyclin D1蛋白。该研究表明，致癌因子长期作用于呼吸道上皮组织，会导致广泛的细胞DNA损伤，这其中可能有癌基因的激活和抑癌基因的失活，经过长期的突变累积，最终发展为恶性肿瘤。而在这一过程中，不同位置的组织细胞可能发生不同的基因突变，从而形成所谓具有独立不同克隆来源的多原发癌，而各个癌灶就表现出具有自身特异性的基因表型。

### 2.2 人口老龄化

随着医疗卫生水平的提高，人的平均寿命得以延长，与此同时也为肿瘤的发生发展提供了时间长度。外科手术技术的不断进步、放疗化疗等多学科治疗方案的不断优化，使肿瘤患者的总生存期增加，第二原发肿瘤发病率随之增加。有研究^[16]^称，54岁-64岁之间的肺癌患者中只有5%-12%有既往肿瘤病史，而在80岁以上的癌症患者中其比例可达12%-26%。

### 2.3 家族遗传因素

Li^[17]^发现MPLC患者具有家族遗传倾向，具有肿瘤家族史的肺癌患者发生第二肺癌甚至第三肺癌的机率明显高于没有家族遗传史的患者；同时该项研究还指出，具有肿瘤家族史的肺癌患者发生第二原发癌的机率升高9倍。

### 2.4 医源性因素

随着医疗卫生条件的改善，高分辨计算机断层扫描（computed tomography, CT）、正电子发射型计算机断层显像（positron emission tomography-computed tomography, PET-CT）及荧光内镜等先进诊断技术的应用，使肺癌在早期得以诊断和治疗，提高了患者初发癌的疗效，生存期延长，第二原发肺癌的检出率也相应增加。随着肿瘤综合治疗手段的进展，近70%的肿瘤患者要接受放疗或化疗，一方面使患者原本受损的免疫力进一步下降，降低了患者的肿瘤防御能力；另一方面放疗、化疗本身也具有远期致癌可能。

## 3 MPLC的诊断

1975年，Martini等^[18]^提出了诊断多原发肺癌的临床标准（M-M标准），其要点包括：sMPLC：①各癌灶之间有一定距离，且彼此孤立；②各癌灶组织学类型不同；③组织学类型相同时：各癌灶位于不同的解剖区域（不同的肺段、肺叶，或不同侧），由不同的原位癌起源，各癌灶共同的引流区域无癌肿累及且确立诊断时无肺外转移。mMPLC：①各癌灶组织学类型不同；②组织学类型相同时：无瘤间期至少2年，或均由不同的原位癌起源，或第二原发癌位于不同肺叶或不同侧肺时，但各癌灶共同的淋巴引流部位无癌肿，确立诊断时无肺外转移。可见，癌灶的形态、位置和共同淋巴通道是否浸润等病理学特征为诊断MPLC的要点。由于该标准简便、可操作性强，一直以来为临床医师和病理学家广泛接受和采用。

进入21世纪后，随着基因与分子生物学技术的不断发展，M-M标准也被不断补充和改进。美国胸科医师协会（American College of Chest Physicians, ACCP）先后于2003年、2007年和2013年对M-M标准进行了补充，2013年ACCP^[19]^关于MPLC的诊断要点如下：sMPLC：①各癌灶组织学类型不同；②各癌灶具有不同的分子遗传特征；③各癌灶由不同原位癌起源；④各癌灶组织学类型相同时，各癌灶位于不同肺叶且无纵隔淋巴结转移及无全身转移。mMPLC：①各癌灶组织学类型不同；②各癌灶具有不同的分子遗传特征；③各癌灶由不同原位癌起源；④组织学类型相同时，无全身转移且两病灶间隔不少于4年；⑤若组织学类型相同，无纵隔淋巴结转移及无全身转移，但两病灶间隔时间≥2年且 < 4年时，不能确定是MPLC还是转移。此外，该指南还定义了肺癌的卫星灶（病灶位于同一肺叶内，组织学类型相同，并且没有远处转移）和肺癌的肺内转移灶（组织学类型相同，伴纵隔淋巴结转移，或有远处转移，或发病间隔时间 < 2年）。相对于M-M标准，ACCP关于MPLC的诊断标准增加了分子遗传学特征，同时兼顾了MPLC与转移的鉴别。鉴于MPLC以肺腺癌居多^[11]^，2013年ACCP推荐利用肺腺癌的组织学亚型鉴别MPLC与肺内多发转移，还提出了另外一种鉴别手段--分子遗传学分析，即利用特异的分子标志物或基因突变位点加以鉴别。

虽然有了上述诊断标准，但是临床上对MPLC的诊断依旧是个难题。仅仅根据肺癌病灶位置和大小来鉴别MPLC和肺内转移是不完善的，因此ACCP指南建议，MPLC与肺内转移癌的鉴别，需要综合考虑其临床特点及影像学特征。较为可靠的鉴别方法是分子遗传学分析，利用分子生物学技术对特异性分子标记物或突变位点进行检测和分析，确定两癌灶的异源性，但由于经济效益等原因，这些方法很少应用于临床实践中。无论是M-M标准还是ACCP指南，只是作为临床医生参考。MPLC需要多学科综合诊断，诊断时需要综合考虑组织学类型、遗传学特点、影像学特征以及临床表现等加以诊断。

## 4 MPLC的鉴别诊断

在临床处理策略的选择上，需要将MPLC与肺内转移癌、肺癌复发相鉴别。根据ACCP指南，当两癌灶组织学类型不同时为多原发，但组织学类型相同时鉴别难度较大。在临床实践中，我们首先是借助于胸部HRCT进行鉴别：MPLC大多表现为孤立的、边缘不光整、密度欠均匀的类圆形结节影，可伴有毛刺和分叶征，肿瘤倍增时间较长。转移癌则常表现为边缘光滑、密度较均匀的球形阴影，少见毛刺及分叶征，多位于肺周围的浅表层，肿瘤倍增时间较短。

MPLC与转移癌在克隆起源上存在本质差异，因此可以从分子及基因水平来鉴别MPLC与转移癌。诸多研究也表明，基因分析在MPLC与肺癌肺内转移鉴别过程中具有重要意义。早在1995年，Antakli等^[2]^就引入了DNA倍体类型差异和*p*53基因突变的同源性进行原发癌和转移癌的鉴别诊断，但这种检测敏感性相对较低。Wu等^[20]^提出，对于病理类型相同的MPLC患者，可以对病灶组织的*p*53基因（外显子5-8）和*EGFR*基因（外显子18-22）的突变情况进行检测和联合分析，以了解多病灶的克隆来源是否相同。van Rens等^[21]^对31例MPLC患者（64个癌灶）进行*p*53基因突变检测分析发现，21例患者癌灶之间存在不同的*p*53突变位点或突变模式，2例存在相同的突变方式而被诊断为转移癌；另8例患者因各个肿瘤灶均无*p*53突变或DNA标本不达标而未被确诊，表明*p*53突变方式分析可以作为鉴别MPLC与转移癌的一个手段。

近年来，一些新技术如染色体位点杂合性缺失（loss of heterozygosity, LOH）分析、比较基因组杂交技术（comparative genomic hybridization, CGH）和二代测序等手段逐渐用于探索多发肺癌病灶的克隆起源。Froio等^[22]^对一例sMPLC患者的3个癌灶进行了40个染色体位点的杂合性缺失（loss of heterozygosity, LOH）分析，结果表明，3个癌灶的LOH相互存在差异，这种差异表现为：①不同肿瘤灶表现为不同的染色体位点突变；②不同肿瘤灶存在相同的染色体突变位点，但突变方式不同；该结果说明这3个肿瘤灶彼此独立，属于多克隆起源，为多原发肺癌。Huang等^[23]^对多原发肺癌及转移癌肿瘤的12个基因位点的LOH进行分析，发现原发性肺癌和转移癌有着近乎一致的杂合性缺失，而目前临床诊断的大多数同时性及异时性MPLC各癌灶杂合性缺失形式表现出差异性。Arai等^[24]^应用比较基因组杂交技术（array comparative genomic hybridization, aCGH）检测了12例同时双肺癌患者两个肿瘤的DNA拷贝数变化的一致率，以鉴别多原发肺癌与转移癌，当一致率>50时考虑为转移癌，反之为多原发肺癌；结果显示病理诊断与分子诊断的一致率达83%（10/12）。Shen^[25]^对MPLC和肺内转移癌的癌细胞基因组的6个微卫星多态性位点检测分析，进而区别二者的克隆起源关系。

除了基因水平外，亦有在蛋白质分子水平探索MPLC与肺内转移癌的区别的研究。Ono等^[26]^研究，分析了4个癌症相关蛋白（p53、p16、p27和c-erbB-2）的表达差异，进而对MPLC与转移癌加以鉴别。Kadara^[27]^通过免疫组化分析CK19、p53、CEA、Hup-1、PE-10和Ki-67蛋白表达，对临床诊断为MPLC的患者及转移癌患者进行二次诊断，结果显示，7例临床标准诊断为多原发癌中的6例及8例临床诊断为转移癌中的5例被重新诊断为肺多原发癌。以上研究说明了临床诊断标准存在的不足。近来也有学者提出采用二代测序及分析基因组重排破裂点（break points of genomic rearrangement）以鉴别MPLC与转移癌^[28]^。

## 5 治疗的选择和预后

目前，关于多原发肺癌的治疗还没有权威指南，尽管如此，仍有一些基本的公认原则：①在无手术禁忌证的情况下尽可能手术治疗；②尽可能完整有效地切除肿瘤；③尽可能多地保留健康肺组织；④术后应采取多学科综合治疗，以提高生存率。

Tsunezuka^[29]^通过30余年的随访研究发现，对于双侧多原发肺癌，积极的手术效果极佳，根据病灶特征，可以选择袖形切除术或者局限性切除。Kim^[30]^研究认为，对于可手术切除的NSCLC伴其他肺叶GGO的患者，若GGO≥8 mm，则应手术切除；反之则影像学密切随访，若病灶增大或出现实性变，则行手术切除。Trousse^[31]^经过长期的研究指出，肺段切除术/肺叶切除术+淋巴结清扫术是能够使MPLC患者获得最好预后的手术方案，同时还指出术后的辅助治疗可以使患者获益。经过多中心的长期临床研究，关于MPLC的手术方式达成了一些共识：对于异时性多原发肺癌，首发病灶一般按照常规的NSCLC的手术方案进行治疗；第二原发癌尽可能地行局部切除，局部切除具有低手术风险及低肺功能损失的优势，但若病灶>3 cm、分期较晚者，建议行肺叶切除术+淋巴结清扫术。对于同时性MPLC病灶位于单侧肺同一叶者，多采用肺叶切除；同时性病灶位于单侧肺不同叶者，一般较大病灶所在的肺叶行肺叶切除，小病灶采取肺楔切，若两病灶均较小，则行不同肺叶楔切；对于病灶位于双侧肺的MPLC，多采用分期手术，2次手术相隔1个月左右，遵循的手术原则是：先切除中央型、进展较快、病灶较大或伴有纵隔、肺门淋巴结转移的病灶，后切除周围型、进展较慢、病灶较小或无淋巴结转移的病灶，即先切除对预后影响较大、分期较晚的病变。

Takamochi^[32]^的研究表明，手术治疗MPLC患者3年和5年生存率分别高达82.1%和77.3%，明显较肺内转移癌预后好；Yang^[33]^对101例接受手术治疗的双侧多原发肺癌患者进行了随访研究，3年和5年生存率分别为84.5%和75%，该研究还指出，对于临床分期属于Ⅰ期的双侧原发肺癌采取局限性切除，并不影响5年生存率；来自日本的一项研究^[7]^报道术后5年生存率高达87.0%。而早些年的报道中，MPLC的5年生存率仅为30%-50%^[34, 35]^，以上表明早期的筛查、微创外科技术的发展以及术后辅助治疗的应用使患者生存期明显延长。

Tsunezuka等^[29]^研究报道同时性MPLC预后优于异时性MPLC，但Jiang的*meta*分析^[36]^认为二者的预后无明显差异。Chang等^[12]^的研究通过单因素分析发现，淋巴结转移、血管受累、组织学类型与预后有关，多因素分析提示只有淋巴结转移是影响其预后的独立因素，患者有无淋巴结转移5年生存率分别为15.5%和52.5%。另一项研究^[31]^对125例同时性MPLC单因素分析发现：吸烟、高Charlson指数、较差的肺功能、全肺切除的患者预后较差；双侧病变、病灶位于同一肺叶、无淋巴结受累者预后较好；多因素分析提示：较差的肺功能、未手术治疗、全肺切除是预后独立危险因素，但日本的研究^[7]^提示双侧肿瘤患者预后不良。此外有研究^[37]^表明p53和HER2蛋白过度表达提示多原发肺癌预后不良，是影响多原发肺癌患者预后的独立因素。

对于分期较晚或难以耐受手术的患者，可以选择放、化疗等其他辅助治疗手段。有研究^[38]^报道，对于无法进行局部切除的中央型MPLC，为了保存较多的肺功能，可以采用立体定位放疗（stereotactic body radiation therapy, SBRT），局部控制率可达95.2%，相关并发症发生率仅为22.2%；分子靶向治疗对于基因突变的患者效果较好。此外，也有利用光动力疗法（photodynamic therapy, PDT）、免疫治疗等其他手段治疗MPLC的报道。

## 6 总结与展望

近年来，MPLC的发病率呈逐渐升高趋势，需要给予重视。“区域癌化”为大多数学者所接受，发病机制的研究为MPLC的预防、诊断及治疗提供了理论基础。分子生物学技术的应用将MPLC的诊断及鉴别诊断提升到了一个新的水平，但MPLC的诊断及鉴别诊断仍需要综合考虑病灶的组织学类型、遗传学特点、影像学特征以及临床表现。在治疗方式的选择上，应以积极的手术为主，手术应遵循“尽可能完整有效地切除肿瘤，尽可能多地保留健康肺组织”的原则，术后予以适当的辅助治疗，以延长患者生存期。

临床医师应提高对多原发肺癌的认识，以免因缺乏认识而使部分患者失去手术机会。对于术后患者，应做好随访工作，以便及时发现可能的第二原发癌^[39]^。现有的分子遗传学手段存在操作费时、灵敏度不高、价格昂贵等缺点，很难在临床实践中大规模应用，探索更为灵敏、经济的特异遗传分子标志物以鉴别MPLC与肺内转移癌显得颇为重要。可以预期，随着MPLC研究的广泛展开和不断深入，将会有更加合理规范的指南以引导临床实践。
